# An experimental study using a sexual strategies explanation to reduce homophobia toward gay men among lay people and healthcare professionals in China

**DOI:** 10.3389/fpsyg.2023.1143584

**Published:** 2023-04-20

**Authors:** Qianhui Gao, Jan Antfolk, Pekka Santtila

**Affiliations:** ^1^School of Psychology and Cognitive Science, East China Normal University, Shanghai, China; ^2^Faculty of Arts and Sciences, New York University Shanghai, Shanghai, China; ^3^Faculty of Arts, Psychology, and Theology, Åbo Akademi University, Turku, Finland

**Keywords:** homophobia, gay men, evolutionary psychology, sexual strategies theory, healthcare professional, gay affirmative practice

## Abstract

**Introduction:**

Homophobic discrimination and stigmatization, especially from healthcare professionals, are important stressors for gay men. Homophobia may be partly rooted in seeing some gay men having casual sex and many sexual partners as a signal of mental problems. Sexual Strategies Theory (SST) suggests that such sexual behavior is a result of different sexual strategies men and women tend to adopt and is unrelated to sexual orientation *per se*. This study aimed to investigate (1) the effectiveness of providing an SST explanation for gay men’s sexual behavior in reducing homophobia among both lay persons and healthcare professionals; (2) differences in homophobia between healthcare professionals and lay people and also between medical and non-medical professionals.

**Methods:**

The main analyses included 492 heterosexual participants recruited online via Chinese social media and sample services in 2021. Of these, 227 were healthcare professionals (128 medical, 99 non-medical) and 265 were lay people. The participants were randomly assigned into an experimental group given the SST explanation (*n* = 126), an active control group given a Minority Stress (MS) explanation (*n* = 184), and a control group (*n* = 182). After the manipulation, homophobia, knowledge about homosexuality, professional homophobic attitude, gay affirmative practice, and contact with gay men were assessed.

**Results:**

The results of factor analysis suggested dividing homophobia into Oppressing Homophobia (Oppressing HP) describing believing that gay men should have fewer rights and Pathologizing Homophobia (Pathologizing HP) describing believing that the sexual behavior of gay men is a signal of mental problem. Importantly, the SST explanation reduced Pathologizing HP while the MS explanation reduced Oppressing HP. Healthcare professionals reported more Oppressing HP than lay people, and medical professionals conducted less gay affirmative practice than non-medical professionals.

**Conclusion:**

An SST explanation can potentially reduce some aspects of homophobia among both healthcare professionals and lay people. Also, worryingly, Chinese healthcare professionals, especially medical professionals, reported more homophobia than lay individuals.

## 1. Introduction

Discrimination is one of the major causes of the mental and physical health disparity in gay men. Homophobic attitudes may partly stem from negative attitudes toward some gay men engaging in casual sex and having multiple sexual partners and beliefs that this signals mental problems. Traditionally, the Minority Stress (MS) perspective has been used to both explain this and to reduce homophobia. However, this explanation can be seen to confirm a negative view of sexual behavior making it potentially problematic. Here, we tested a new approach to reducing homophobia by providing the participants with a Sexual Strategies Theory (SST)-based explanation of the sexual behavior of gay men. Given that healthcare professionals play an important role in promoting gay men’s health, this is a particularly relevant group to focus on. The current study investigated homophobia in healthcare professionals using lay individuals as controls.

Homophobic discrimination is one of the major stressors for gay men having negative mental and physical health consequences. Homophobia refers to devalued status of non-heterosexual behaviors, identities, interactions, or communities and the hostile attitude against sexual minority groups generated from this knowledge (Herek, 1988, 2007, 2009; Herek et al., 2009; Fraïssé and Barrientos, 2016). A typical component of discrimination is stigmatizing gay men as promiscuous (e.g., having more frequent sex and having multiple sexual partners; Rice et al., 2021) and pathologizing it as a signal of mental problem (Kraus et al., 2016; Pinsof and Haselton, 2016; Pinsof and Haselton, 2017; Klein et al., 2019). Salvati and Koc (2022) emphasized the importance of reducing the homophobia of the public and improving the social status of LGBTQ+ people.

One approach that has been used to understand the sexual behavior of gay men is the Minority Stress (MS) perspective which suggests that gay men engage in more casual sexual activities and having more sexual partners are consequences of this stress (Kashubeck-West and Szymanski, 2008; Emlet et al., 2017; Parrish et al., 2019; Tan, 2019). For example, some gay men may use sex to release the stress caused by discrimination (Kashubeck-West and Szymanski, 2008), while some gay men engage in risky sexual behaviors because they do not have access to HIV prevention programs (Philbin et al., 2018). Also, because of discrimination gay men may experience difficulties in forming and maintaining long-term relationships. In fact, some educational programs describe how the sexual behaviors of gay men are shaped by the discrimination they receive in order to reduce homophobia (Godfrey et al., 2006), and some healthcare professionals use this view as a starting point to help gay men regulate sexual behaviors. However, an argument can be made that this view actually confirms the sex-negative and biased idea that gay men being sexually active is problematic and requires regulation. It reinforces the perception that some gay men engaging in casual sexual activities are “deviant” and if they did not experience discrimination and were psychologically balanced, they may not engage in as many sexual activities.

This information may cause problems because it may enhance the stigmatization of gay men by assuming that their behaviors are inherently linked to discrimination and minority stress, rather than taking a more positive view of their sexual behaviors. Also, it may risk preventing healthcare professionals from effectively providing information and education to facilitate gay men exploring and expressing their sexuality and sexual preferences freely and safely based on their own desires, and making sex a positive and beneficial experience.

An alternative perspective, based on the Sexual Strategies Theory (SST), suggests that gay men engage in more casual sex and have more sexual partners as a result of the differences in potential costs and benefits of sexual activities between men and women, unrelated to sexual orientation *per se*. Women have higher obligatory costs than men in case sexual activity results in conception, including pregnancy and, subsequently, giving birth and breastfeeding. In men, sexual activity results in less obligatory parental investment (Trivers, 1972). In addition, the maximum number of offspring that a man can have is unlimited, while for women it is limited to only a few. This means that having additional sexual partners may increase the number of offspring for men without much investment whereas for women this is not true. The large differences in parental investment and potential benefits of sexual activity have been hypothesized to have resulted in women being more sexually restrictive than men (Trivers, 1972). Consequently, in heterosexual relationships, men’s interest in sexual activities is, according to theory, limited by women’s more restrictive attitudes. However, in a potential sexual encounter between men, all partners are relatively more interested in sexual activities, thus, actual sexual activities are more likely to take place (Buss, 2016). Under this explanation, the sexually unrestricted behavior observed in gay men does not rely on sexual orientation. Instead of seeing gay men being more sexually active as deviant, the SST de-pathologizes it as a natural result of sexual strategy differences between men and women serving an adaptive function and normalizes it as a tendency that all men share regardless of their sexual orientation. Thus, including the content of SST in education and training to reduce the stigmatization and discrimination attached to the sexual behaviors of gay men seems potentially useful.

Discrimination from healthcare providers may decrease the quality of health services the sexual minority individuals receive, or even detach them from the healthcare system or preventive programs (Larsson et al., 2016; Currin and Hubach, 2017; Currin et al., 2018; Philbin et al., 2018; Currin et al., 2020), which can, in turn, reinforce the stigmatization of gay men being problematic. Paradoxically, even though one should expect professionals to be well informed, discrimination against gay men is still widespread in healthcare systems (Smith and Mathews, 2007; Blackwell and Kiehl, 2008; Lin et al., 2021; Kaya and Calpbinici, 2022).

Given that healthcare professionals encounter gay men when they are looking for professional help, these professionals may be especially likely to associate any problems with the patient’s sexual orientation including pathologizing gay men for being “promiscuous” (Rice et al., 2021) compared to heterosexual men due to confirmation bias (i.e., one uses one’s *a-priori* assumption to interpret the observed phenomenon; Frey, 1986; Nickerson, 1998). This, in turn, can shape their attitudes toward gay men in the absence of other contacts with gay men (Smith and Mathews, 2007; Kan et al., 2009; Klein et al., 2019; Elston, 2020). In addition, the professional role of medical professionals is one of the identifying pathologies. Combining this with the fact that being gay used to be a diagnosis until the 1970s in the US (Stoller et al., 1973) while in China, homosexuality was only removed from the list of mental illnesses in the Chinese Classification of Mental Disorders Version 3 (CCMD-3) in 2001, may result in many professionals still having outdated information about and attitudes in relation to gay men. Due to information delays in education (e.g., information from the supervisor, outdated textbooks), there is a chance that even recently trained professionals do not recognize homosexuality as a natural expression of human diversity.

In the healthcare field, previous research emphasizes that specific education and training on sexual and gender minority topics are necessary to address professionals’ own prejudice (Godfrey et al., 2006; Kan et al., 2009; Lin et al., 2021) and to increase affirmative competence and practices (Kocarek and Pelling, 2003; Long and Serovich, 2003; Alderson, 2004; Israel and Hackett, 2004; Matthews et al., 2005; Rutter et al., 2008; Rock et al., 2010; Bidell, 2012; Pereira et al., 2019). The education and training should include components such as social stigma, internalized homophobia, and sexuality (Godfrey et al., 2006).

## 2. Aims and hypotheses

The Minority Stress (MS) explanation may reinforce gay men’s sexual activities as deviant while Sexual Strategies Theory (SST) normalizes and de-pathologizes such behavior. We therefore expected that an SST explanation would reduce homophobia compared to both a MS explanation and no explanation. We also investigated whether this effect was moderated by the gender or professional group of the participants.

In line with previous research in mostly Western settings, we expected being a heterosexual man, having less knowledge about homosexuality, and having less contact with gay men to be associated with more homophobia (Herek, 1988, 2007; Herek and Capitanio, 1996).

Finally, healthcare professionals, especially medical professionals, may still take the pathologizing view of homosexuality due to outdated information and education as well as confirmation bias connecting patients’ sexual orientation to their problems. We therefore expected healthcare professionals to exhibit more homophobia than lay individuals and that medical professionals would exhibit more homophobia than non-medical professionals. We also expected medical professionals to exhibit more professional homophobia and less gay affirmative practice than non-medical professionals.

## 3. Method

### 3.1. Participants

In total, 492 Chinese heterosexual participants were included in the analyzes for the presented study. According to the analysis on the basis of G*power by using an F-test (power of 0.80, and medium effect size of *f* = 0.25), a 3 by 2 between-subject design involving three groups should recruit at least 159 participants. Originally, 629 individuals participated, but the data of 70 participants were removed due to not passing the attention check. Also, the data of 67 non-heterosexual participants were excluded to manage the impact of sexual orientation on the results. Of the 429 heterosexual participants, 227 were healthcare professionals and 265 were lay people.

The participants were recruited in China via social media and via the sample service of Credamo (www.credamo.com, a Chinese online questionnaire platform) and using snowball sampling of healthcare professionals. The healthcare professionals from Credamo and social media were encouraged to share the study with their colleagues and professional friends.

Healthcare professionals were doctors/physicians (*n* = 41), nurses (*n* = 79), medical students (*n* = 8), psychologists (*n* = 4), psychology students (*n* = 2), psychotherapists (*n* = 63), psychiatrists (*n* = 6), marriage and family therapists (*n* = 3), social workers (*n* = 14), and other occupations related to healthcare (e.g., psychology teachers, *n* = 7).

The healthcare professionals were further divided into medical healthcare professionals and non-medical healthcare professionals. Medical healthcare professionals were doctors, nurses, and medical students; non-medical healthcare professionals were psychologists, psychology students, psychiatrists, marriage and family therapists, social workers, psychotherapists, and other occupations related to healthcare.

A total of 492 heterosexual participants were randomly assigned to three groups: Sexual Strategies Theory group (SST group), Minority Stress group (MS group), or Control group. The assignment of participants to groups was carried out randomly. Of the healthcare professionals, 49 were assigned to the SST group, 85 were assigned to the MS group, and 93 were assigned to the Control group. Of the non-professionals, 77 were assigned to the SST group, 99 were assigned to the MS group, and 89 were assigned to the Control group.

The demographic information of the participants is shown in Table 1. Of the healthcare professionals, 44.5% (*n* = 101) were aged from 26 to 30; 42.3% (*n* = 96) were men, while 57.7% (*n* = 131) were women; more than half had a Bachelor degree or above; 56.4% (*n* = 128) were medical professionals, while 43.6% (*n* = 99) were non-medical professionals.

**Table 1:** Demographic and grouping information.

Group and characteristic	Professional (*n* = 227)		Non-professional (*n* = 265)	
	*n*	%	*n*	%
Intervention group				
AE	49	21.6	77	29.1
MS	85	37.4	99	37.4
Control	93	41.0	89	33.6
Age				
18 ~ 25	51	22.5	56	21.1
26 ~ 30	101	44.5	80	30.2
31 ~ 40	62	27.3	116	43.8
41 ~ 50	10	4.4	10	3.8
51 ~ 60	3	1.3	3	1.1
Gender				
Male	96	42.3	94	35.5
Female	131	57.7	166	62.5
Nonbinary	0	0	5	1.9
Degree				
Middle school or under	1	0.4	1	0.4
High school	18	7.9	17	6.4
Bachelor	177	78.0	207	78.1
Master or above	31	13.7	40	15.1
Occupation				
Medical	128	56.4		
Nonmedical	99	43.6		

Of the non-professionals, 43.8% (*n* = 116) were aged from 31 to 40; 35.5% (*n* = 94) were men, while 62.5% (*n* = 166) were women, and 1.9% (*n* = 5) identified themselves as nonbinary; more than half had a Bachelor degree or higher.

### 3.2. Instruments

#### 3.2.1. Demographic information

The demographic information included age, gender (1 = *Male*, 0 = *Female*; *Nonbinary participants were not included in analyzes involving gender*), education (1 = *Middle school or under*, 2 = *High school*, 3 = *Bachelor*, 4 = *Master or above*), and sexual orientation (1 = *Heterosexual*, 0 = *Non-heterosexual*). Also, among healthcare professionals, medical and non-medical professionals were separated (1 = *Medical*, 0 = *Non-medical*).

#### 3.2.2. Homophobia

Homophobia was assessed by a 14-item scale modified based on the scale developed by Herek (1988) (See Table 2 for all items). The questions are still widely used to measure attitudes toward gay men among both healthcare professionals and lay people (Baiocco et al., 2021; Rollè et al., 2021). The original scale contained 10 items and the internal consistency reliability was 0.90. Sample items included “4. Male homosexuality is a perversion” and “5. Just as in other species, male homosexuality is a natural expression of sexuality in human men (reverse scoring).” Response options ranged from 1 (strongly disagree) to 5 (strongly agree). We added four items that measured the individuals’ tendency to pathologize the sexual behavior of gay men. The added items were: “11. I feel that the sexual behavior of gay men is a sign of psychological problems,” “12. Gay men should stop having sex with men,” “13. Gay men should feel bad about themselves for having casual sex,” “14. Gay men cannot control their sexual behaviors because it is a kind of mental problem.” The internal consistency reliability of the 14 items was.92.

**Table 2:** Two subtypes of homophobia.

Subtype	Item	Oppressing homophobia	Pathologizing homophobia
Oppressing homophobia	8. Homosexual behavior between two men is just plain wrong.	0.800	0.743
	9. The idea of male homosexual marriages seems ridiculous to me.	0.787	0.688
	2. I think male homosexuals are disgusting.	0.743	0.643
	3. Male homosexuals should not be allowed to teach school.	0.710	0.597
	5. Just as in other species, male homosexuality is a natural expression of sexuality in human men.^*^	0.702	0.406
	10. Male homosexuality is merely a different kind of lifestyle that should not be condemned.^*^	0.678	0.380
	1. Male homosexual couples should be allowed to adopt children the same as heterosexual couples.^*^	0.619	0.363
	7. I would not be too upset if I learned that my son was a homosexual.^*^	0.577	0.515
Pathologizing homophobia	12. Gay men should stop having sex with men.	0.761	0.792
	4. Male homosexuality is a perversion.	0.723	0.753
	11. I feel that the sexual behavior of gay men is a sign of psychological problems.	0.414	0.729
	14. Gay men cannot control their sexual behaviors because it is a kind of mental problems.	0.372	0.703
	6. If a man has homosexual feelings, he should do everything he can to overcome them.	0.529	0.639
	13. Gay men should feel bad about themselves for having casual sex.	0.460	0.469

Scoring was reversed for the starred (^*^) items.

After using a Principal Components analysis to determine that the homophobia items were underpinned by two components, we conducted an Alpha Factoring factor analysis with oblique rotation and found that the 14 items actually measured two subtypes of homophobia that we labeled Oppressing Homophobia (Oppressing HP) and Pathologizing Homophobia (Pathologizing HP). Oppressing HP indicates devaluing non-heterosexual individuals and believing they should have less access to resources and less influence in important fields Pathologizing HP indicates believing homosexuality is a signal of mental problems. The two factors together explained 52% of the variance.

The Structure Matrix in Table 2 shows that the eight items formed the first subtype and the rest of the items formed the second. The internal consistency reliability of the items was 0.89 and 0.84 for Oppressing and Pathologizing HP, respectively. Factor scores created using the regression method were used in analyzes involving these variables.

#### 3.2.3. Knowledge about homosexuality

Knowledge about homosexuality was measured by a 20-item true or false questionnaire created by Harris et al. (1995). The sum of correctly answered questions was calculated and used in the analyzes. The internal consistency reliability of these 20 items was 0.70.

#### 3.2.4. Contact with gay men

The participants were asked if they had in-person contact with anyone who is gay (0 = *No*, 1 = *Yes*). Those who answered “*Yes*” were further asked to indicate how many gay men they knew (1 = *Only 1*, 2 = *2 to 5*, 3 = *5 to 10*, 4 = *more than 10*); how well they knew the gay man that they knew the best (1 = *I know very little about him*, 10 = *I know very well about him*); and how close they were with the gay man they were the closest to (1 = *We are not close at all*, 10 = *We are very close*).

As the items were on different scales, we z-standardized them and then used the mean of the z-standardized scores in the analyzes. The internal consistency reliability of the items was 0.67.

#### 3.2.5. Attitude toward homosexuality among healthcare professionals

Attitude toward homosexuality in the healthcare context was measured by a questionnaire created by Grabovac et al. (2014). The questions could be answered with Yes, No, and Do not know. We gave 1 point to certain answers of specific items and created a summary variable named Professional Homophobic Attitude. The internal consistency reliability of these items was 0.74. The item “Would you feel more comfortable if you did not have to treat LGBT patients” from the original scales was left out from the questionnaire due to a technical mistake. Also, there were three items (Do you think that LGBT people are discriminated against by doctors and that they receive lower quality care; Do you think that LGBT people are more likely to be infected with or carry an STI; Have you ever read LGBT affirmative therapy literature) that were included in the survey but were not used for creating the summary variable because they did not measure the attitude or behaviors of the participants. Table 3 provides details regarding the items included in the summary variable and the scoring.

**Table 3:** Professional homophobic attitude scoring.

Item	Giving 1 point to the answers of
Do you think that LGBT patients should come out to their doctors?	“Do not know” and “No”
Have you ever had an LGBT patient?	
If yes (to item ‘Have you ever had an LGBT patient?’), what were your experiences like?	“mostly negative” and “completely negative”
Do you think that doctors have unpleasant experiences with LGBT patients more often than with heterosexual patients?	“Do not know” and “Yes”
Would your attitude toward a patient change if he/she were to come out to you?	
Do you think that LGBT patients should receive last appointments (in a work day) for treatment?	
Would you, as a future doctor, be scared or apprehensive to meet an LGBT patient?	
Do you think that you should behave differently when dealing with an LGBT patient? (for instance, protect yourself better against infection)	
If a patient came out to you, would you tell that to your colleagues?	
Do you think that LGBT people should be ashamed of their sexual orientation?	
Do you consider homosexuality to be an illness?	
If you had the opportunity, would you refuse to give an injection or draw blood from an LGBT patient?	
Would you feel uncomfortable to have an LGBT colleague?	

#### 3.2.6. Gay affirmative practice of healthcare professionals

Gay affirmative practice was evaluated by a Likert-type scale created based on the work of Crisp (2006). The subjects indicated the frequency of conducting gay affirmative practice (5 = *Always*, 1 = *Never*). Sample items were “I help clients reduce shame about homosexual feelings” and “I help gay/lesbian clients address problems created by societal prejudice.” The internal consistency reliability of the scale was 0.92.

### 3.3. Procedure

The participants were recruited via the Chinese research worker service Credamo^1^ and via snowball sampling in social media.

First, we posted the survey on Credamo. Every Credamo user who saw the survey in the Data Market could participate in the survey. Both healthcare professionals and non-healthcare professionals were recruited via this method. Also, to recruit more healthcare professionals, the snowball sampling method was used. The investigator posted the link to the online study on social media such as WeChat. The healthcare professionals from Credamo and social media were encouraged to share the study with their colleagues and professional friends.

The participants were randomly assigned into three groups: Sexual Strategies Theory Group (SST group), Minority Stress Group (MS group), and Control group.

#### 3.3.1. Group 1: Sexual strategies theory group

In this group, the participants were shown a video that explained why gay men were more likely to have casual sex behavior from an evolutionary psychological perspective based on sexual strategies theory. Then, they were instructed to complete a questionnaire that assessed homophobia, knowledge about homosexuality, and contact with gay men. In addition, healthcare professionals needed to report their attitude toward homosexuality and their condition of gay affirmative practice.

#### 3.3.2. Group 2: Minority stress group

In this group, the participants were shown a video that explained why gay men were more likely to have casual sexual relationships from a minority stress perspective (no mention of an adaptive explanation is made). Then, they were instructed to complete a questionnaire that assessed homophobia, knowledge about homosexuality, and contact with gay men. In addition, healthcare professionals needed to report their attitude toward homosexuality and their condition of gay affirmative practice.

#### 3.3.3. Group 3: Control group

In this group, the participants completed the same questionnaire as the other two groups without watching any video.

See Table 4 for the main features of both interventions. After watching the videos, the participants were asked questions about the videos. For example, “According to this article/video, gay men are more likely to have casual sex because: (a) For gay men, for whom the other person is also a man with equally high interest in casual sexual activity, sexual activity is more likely to also occur; (b) Gay men are more likely to have psychological problems because of minority stress; (c) Gay men want to spread sexually transmitted infections.” In the analysis, we only included participants who answered the questions correctly.

**Table 4:** Main features of both interventions.

Intervention	Features
Sexual strategies explanation	1. Compared to women, there is less obligatory investment attached to the sexual activities of men. Therefore, men are less choosy regarding sexual activities.
	2. Theoretically, men could have an unlimited number of offspring. Casual sexual activity therefore makes more sense for men.
	3. Both gay and heterosexual men are relatively interested in casual sexual activities but heterosexual men’s sexual activity is limited by women’s more restrictive attitudes.
	4. When it comes to two gay men, both may be relatively interested in casual sexual activities. Therefore, sexual activity is more likely to occur.
Minority stress	1. Individuals with internalized homonegativity are less likely to get information and resources about HIV/STIs from the community.
	2. Internalized homonegativity increases self-destructive behavior.
	3. Minority stress impairs the ability to establish same-sex intimate relationships.
	4. Minority stress increase the needs of escapism. Sex provides an opportunity.
	5. Sexual fantasies and behaviors help alleviate the negative emotions caused by minority stress, such as anxiety and depression.
	6. Having sex with a casual partner helps individuals keep their sexual orientation hidden because there are less strings attached.

### 3.4. Statistical analyzes

The statistical analyzes were conducted using SPSS 28. First, descriptive analyzes were conducted to see the main characteristics of the sample. Second, we conducted two univariate ANOVAs to investigate the impact of gender, being professional vs. non-professional, and intervention type on Oppressing HP and Pathologizing HP, respectively. Also, we conducted two additional univariate ANOVAs to investigate the effect of gender, being medical vs. non-medical professional, and intervention type on Oppressing HP and Pathologizing HP, respectively, given that the medical/non-medical was embedded in the professional group. Third, bivariate correlation tests were conducted to investigate the associations among variables. Also, the bivariate correlation tests were repeated only in the control group to confirm the associations in the absence of the interventions.

## 4. Results

The reporting of the results is divided into two parts. First, the effects of gender, professional group, and intervention type on homophobia were described. Second, correlations between the variables were reported.

### 4.1. The effectiveness of the interventions

#### 4.1.1. Oppressing homophobia

**Figure 1:**
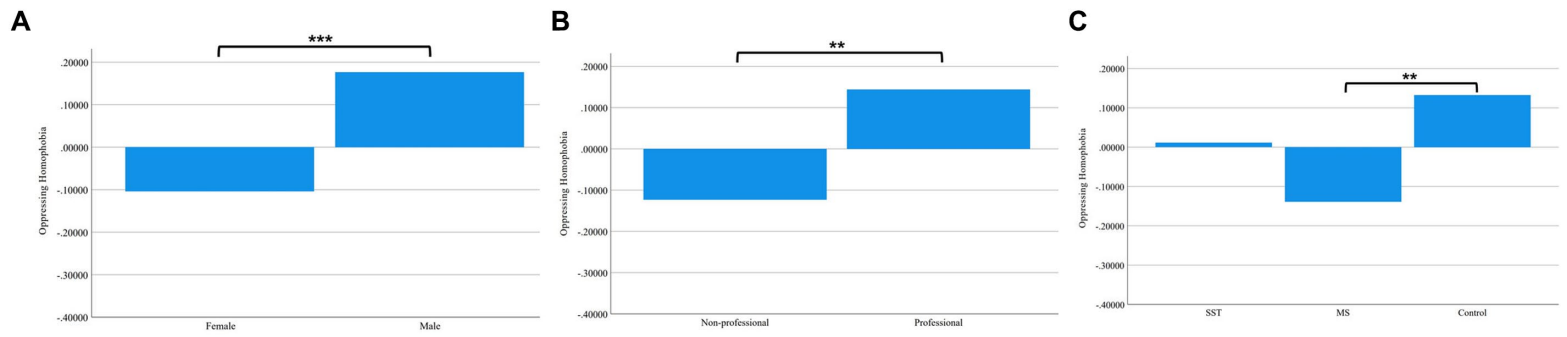
Group Differences in Oppressing Homophobia. **(A)** Differences between Female and Male. **(B)** Differences between Non-professionals and Professionals. **(C)** Differences among Sexual Strategies Theory Group, Minority Stress Group, and Control Group.

To test the effects of intervention, we first conducted two three-way ANOVA analyzes, one for Oppressing Homophobia (Oppressing HP) and one for Pathologizing Homophobia (Pathologizing HP). The independent variables in these two analyzes were gender, professional group, and intervention type. All independent variables had significant main effects on Oppressing HP, but no significant interactions between these factors were found. See Figure 1 for group differences in Oppressing HP. Men had more Oppressing HP than women, *F*(1, 475) = 9.701, *p* = 0.002. Healthcare professionals had more Oppressing HP than the non-professionals, *F*(1, 475) = 6.455, *p* = 0.011. Finally, intervention type also had an impact on Oppressing HP, *F*(2, 475) = 3.574, *p* = 0.029. *Post Hoc* analysis with Duncan’s multiple range test showed that the Minority Stress (MS) group had less Oppressing HP compared to the Control group while the Sexual Strategies Theory (SST) group did not differ from either of the other groups. The results do not support the hypothesis that the SST would reduce homophobia.

#### 4.1.2. Pathologizing homophobia

**Figure 2:**
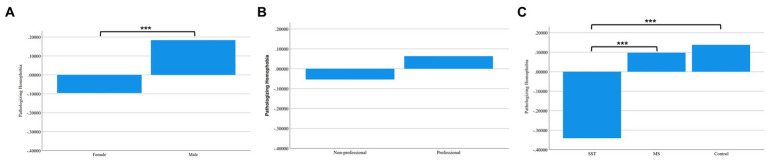
Group Differences in Oppressing Homophobia. **(A)** Differences between Female and Male. **(B)** Differences between Non-professionals and Professionals. **(C)** Differences among Sexual Strategies Theory Group, Minority Stress Group, and Control Group.

Next, we conducted the corresponding analysis for the Pathologizing HP variable. Here, gender and intervention type had significant main effects on Pathologizing HP, respectively, but there was no significant effect of professional group nor were there any significant interactions between the independent variables. See Figure 2 for group differences in Pathologizing HP. Men had more Pathologizing HP than women, *F*(1, 475) = 10.136, *p* = 0.002. The significant main effect of intervention, *F*(2, 475) = 8.904, *p* < 0.001, was due to the SST group having less Pathologizing HP compared to both the MS group and the Control group with the latter two groups not differing significantly from each other as indicated by the Duncan’s multiple range test. The results support the hypothesis that the SST would reduce homophobia compared to the MS explanation and no explanation.

#### 4.1.3. The effectiveness of the intervention in medical and non-medical professionals

Next, we limited our analyzes to the professional group only given that the medical vs. non-medical professional difference was nested within the professional group variable. We then conducted two three-way ANOVAs for the two homophobia variables, respectively. When testing the effect of gender, being a medical vs. non-medical professional, and intervention type on Oppressing HP and Pathologizing HP, we found that gender had a significant effect on both Oppressing HP *F*(1, 215) = 9.50, *p* = 0.002 and Pathologizing HP *F*(1, 215) = 4.57, *p* = 0.034. Neither the effects of medical vs. non-medical professional nor the effects of intervention type were significant in these two analyzes. Further, no interaction effects were found to be significant. These results suggest that being a medical vs. non-medical professional did not moderate the intervention effect.

### 4.2. Factors associated with homophobia

#### 4.2.1. Correlations

See Table 5 for factors associated with homophobia. When including all participants in the analyzes, being male, having less knowledge about homosexuality, and conducting less gay affirmative practice were associated with both more Oppressing HP and Pathologizing HP; being a healthcare professional and being a medical professional were associated with having more Oppressing HP; being older and having less contact with gay men were associated with having more Pathologizing HP. Also, Oppressing HP and Pathologizing HP were positively correlated.

**Table 5:** Correlations between the characteristics of participants and homophobia.

Variable	*M*	SD	1	2	3	4	5	6	7	8	9	10
1. Age	2.26	0.89	-									
2. Gender	0.39	0.49	−0.007	-								
3. Professional	0.46	0.50	−0.090^*^	0.063	-							
4. Medical	0.56	0.50	−0.107	0.141^*^	.c	-						
5. Knowledge	11.52	2.40	−0.186^***^	−0.049	−0.128^**^	−0.226^***^	-					
6. Contact with gay men	−0.47	0.61	0.003	−0.049	0.080	0.049	−0.102^*^	-				
7. Gay affirmative practice	3.31	0.70	0.010	−0.215^**^	.c	−0.276^***^	0.153^*^	0.031	-			
8. Oppressing homophobia	0.00	0.94	−0.060	0.146^**^	0.142^**^	0.133^*^	−0.212^***^	−0.081	−0.441^***^	-		
9. Pathologizing homophobia	0.00	0.89	0.147^**^	0.153^***^	0.065	0.019	−0.347^***^	−0.191^***^	−0.160^*^	0.125^*^	-	
10. Professional homophobic attitude	4.17	2.75	−0.066	0.032	.c	0.092	−0.371^***^	0.014	−0.419^***^	0.620^***^	0.331^***^	-

1 = men, 0 = women; 0 = non-professional, 1 = professional; 0 = non-medical, 1 = medical.

A higher score of Sex knowledge scale indicates more knowledge; a higher score of Contact with MSM indicates having more contact with gay men; a higher score of Gay Affirmative Practice indicates conducting more gay affirmative practice; higher scores of Oppressing homophobia, Pathologizing homophobia, and Professional Homophobic Attitude indicate higher levels of homophobia; .c = Cannot be computed because at least one of the variables is constant.

^*^p < 0.05; ^**^p < 0.01; ^***^p < 0.001.

Table 5 also shows differences between professionals and lay people, as well as between medical and non-medical professionals. Being a healthcare professional was associated with having less knowledge about homosexuality and having more Oppressing HP. Being a medical professional was associated with having less knowledge about homosexuality, conducting less gay affirmative practice, and having more Oppressing HP. Among healthcare professionals, having less knowledge about homosexuality was associated with conducting less gay affirmative practice and having more professional homophobic attitude. Also, conducting less gay affirmative practice, having more Oppressing HP and Pathologizing HP were associated with having more professional homophobic attitude.

Table 5 also shows that being older was associated with being non-professional and having less knowledge about homosexuality; being male was associated with being a medical professional and conducting less gay affirmative practice. Surprisingly, having less knowledge about homosexuality was associated with having more contact with gay men. However, this relationship was specific to healthcare professionals and did not apply to the non-professionals.

#### 4.2.2. Limiting analyzes to the control group

We also repeated the correlation tests only in the control group to investigate whether the above correlation and group differences were impacted by the interventions.

Being male was associated with Pathologizing HP, medical professionals conducted less gay affirmative practice, less gay affirmative practice was associated with more Oppressing HP and more professional homophobic attitude, more Oppressing HP was associated with more Pathologizing HP and more professional homophobic attitude, more Pathologizing HP is associated with more professional homophobic attitude. These correlations confirm the pattern observed in the whole sample.

## 5. Discussion

In the current study, we explained why some gay men engage in more casual sex and have more sexual partners using the Sexual Strategies Theory (SST) to lay people and healthcare professionals, then measured if this explanation reduced homophobia compared to providing them with a Minority Stress (MS) explanation or no explanation of this phenomenon. We also investigated differences in the level of homophobia in lay people and healthcare professionals and explored correlated factors.

### 5.1. The effectiveness of the interventions

Our preliminary analyzes indicated that the homophobia items in the current study could be divided into two types: Oppressing Homophobia (Oppressing HP) and Pathologizing Homophobia (Pathologizing HP). Oppressing HP indicates devaluing non-heterosexual individuals and believing they should have less access to resources and less influence in important fields. An example item is “The idea of male homosexual marriages seems ridiculous to me.” In contrast, Pathologizing HP indicates believing homosexuality and especially homosexual sexual behavior to be a signal of mental problem. An example was “I feel that the sexual behavior of gay men is a sign of psychological problems.” Given our theoretical framework, it would seem logical to expect that the Sexual Strategies Theory (SST) explanation would be particularly effective for the latter homophobia subtype.

Traditionally, information and education from a Minority Stress (MS) perspective have been provided to lay people and healthcare professionals to reduce homophobia (Meyer, 2003; Irwin, 2007; Kashubeck-West and Szymanski, 2008; Philbin et al., 2018). In the current study, compared to the control group, MS in fact reduced Oppressing HP. Even though this was not a hypothesized effect given that our *a priori* hypotheses only concerned homophobia as a whole, the finding is not surprising. By highlighting the impact of discrimination on the mental health and behavior of sexual and gender minority individuals, the MS perspective can increase understanding and empathy for these individuals, leading to reduced homophobia. By recognizing the harmful effects of discrimination and stigma, individuals may become more motivated to take action to promote equality and reduce discrimination, which can lead to a more inclusive and accepting social environment (Herek, 2007).

However, the MS explanation did not reduce Pathologizing HP. This is in line with our thinking. The MS explanation does not de-pathologize gay men’s sexual behaviors as such and can arguably be viewed as sex-negative. It suggests that gay men engaging in more sexual activities is “deviant” caused by stress. This could contribute to the prejudice against gay men by assuming their behaviors are primarily influenced by discrimination and minority stress, rather than recognizing it as a natural aspect of human sexual preferences.

Importantly, the Sexual Strategies Theory (SST) reduced Pathologizing HP compared to both the MS and the control group. This is in line with our expectations. SST emphasizes that gay men are more sexually active than other sexual orientation groups as a result of the different mating strategies men and women tend to adopt, which is unrelated to sexual orientation *per se*.

Compared to women, sexual activities can bring men unlimited benefits without much cost, thus, both heterosexual men and gay men are supposed to be interested in sex. Heterosexual men’s sex interests are limited by women’s more restrictive attitudes. However, when it comes to gay relationships, given that all parties to a relationship are less restricted in sexual partner selection, then actual sex is more likely to happen (Trivers, 1972; Buss, 2016). It seems that sharing this perspective with people may reduce their homophobia to the extent that it relates to sexual behavior. The sexual strategies theory view gay man being more sexually active as a natural sexual inclination that both heterosexual men and gay men have due to the process of evolution. With this explanation, individuals may be less likely to stigmatize or pathologize gay men for their sexual behavior. Also, it may give more space to explore and express sexuality and sexual preference freely and safely.

Interestingly, we found that both SST and MS can be effective in reducing homophobia albeit the impact is on different aspects of this phenomenon. Thus, it may be useful to provide both theories in training. However, so far, training programs aimed at reducing homophobia have not included the SST perspective. This is an important omission. Also, more attention needs to be paid to the potential stigmatizing effects of the MS perspective.

The intervention seemed to work equally in both the non-professional and professional groups. There were also no interactions between the participants being medical vs. non-medical professionals and the intervention even though these analyzes may have been underpowered as they were restricted to only part of the sample. Even though professionals usually see gay men in clinical settings, the intervention was able to reduce Pathologizing HP. It provides evidence that we need to provide education on SST to professionals to reduce homophobia.

### 5.2. Homophobia in lay people and healthcare professionals

As could be expected from previous studies, being male and having less knowledge about homosexuality were associated with both Oppressing HP and Pathologizing HP, while being older and having less contact with gay men were associated with having more Pathologizing HP. Also, Oppressing HP and Pathologizing HP were positively correlated. Nowadays, homosexuality is more visible to the public. However, the public had rather little information or even false information about homosexuality in the past: same-gender attraction and sexual behaviors were a clinical diagnosis until the 1970s (Stoller et al., 1973), and stereotypes of gay men were widespread in social media (e.g., gay men are weak and effeminate; Blashill and Powlishta, 2009; van Meer and Pollmann, 2021). Herek (1988) revealed that adherence to traditional views about gender roles was associated with more homophobia. Salvati et al. (2020) also underscored the significant role of gender stereotypes in contributing to sexual prejudice. Older people were more likely to be misled by outdated information. Men may manifest higher levels of homophobia as a strategy to avoid being labeled “gay” (Herek, 2007) or to defend against masculinity threats (Glick et al., 2007). For the reasons above, older people may be more likely to have little or outdated information about gay men. This might shape homophobia, and in turn, decrease peoples’ contact with gay men.

Also, the results showed that healthcare professionals had more homophobia than the lay people. Furthermore, medical professionals had more homophobia and conducted less gay affirmative practice than non-medical professionals. Our hypothesis was supported. Having more contact with gay men and learning more about homosexuality from these interactions may be an important way to reduce stereotypes about gay men and reduce homophobia (Herek, 2007). The motivation of lay people to reduce homophobia is often based on the need of building good relationships with their gay friends or family members (Herek and Capitanio, 1996; Herek, 2007). Thus, they are more likely to have positive experiences with gay men. However, the motivation for healthcare professionals to regulate homophobia is usually based on the need of providing services to gay men in their workplaces (Murphy et al., 2002). Naturally, clients who seek professional help usually had more obvious problems that may be associated with their identity (otherwise, the patient may not “come out” to the professionals; Larsson et al., 2016; Currin et al., 2018), and this may, in turn, may make medical professionals build a mistaken connection between some pathological conditions (such as having sexually transmitted diseases) and being gay (Rice et al., 2021). Even though most healthcare training programs require courses on diversity, the information and training they provide are usually not sufficient enough to prepare the students to work with sexual minority clients/patients after graduation (Godfrey et al., 2006; Irwin, 2007). In China, most mental healthcare professionals never or rarely received any education or training regarding sexual minority topics (Liu et al., 2017). For these reasons, healthcare professionals, especially medical professionals, may show more homophobia.

Surprisingly, we found having less knowledge about homosexuality was associated with having more contact with gay men. This relationship was specific to the professionals and did not apply to the non-professionals. A possible explanation is that healthcare professionals have more opportunities to interact with gay men than lay people and these contacts can be interpreted in a pathologizing manner.

### 5.3. Limitations and future studies

All the measures of the presented study were conducted after the intervention. To address this limitation, we repeated the ANOVA analyzes only in the control group in which no intervention was conducted. The patterns shown in the control group were also observed in the whole sample. However, the sample size in the control group is relatively small. Another limitation of the current study is that it only investigated homophobia and the effectiveness of intervention in relation to gay men. In future studies, it is important to extend results to, for example, bisexual men (Salvati and Koc, 2022).

### 5.4. Implications

Education about homosexuality is needed to reduce homophobia in both lay people and healthcare professionals. Besides providing information about how discrimination from society caused negative behavioral and health outcomes of gay men, providing information about how their active sexual behaviors might be the consequence of sexual strategies that heterosexual men also tend to favor is important as well—as it can help to de-pathologize those behaviors.

The results suggest that medical schools and clinical psychology training programs should provide the Sexual Strategies Theory (SST) in addition to explaining the MS perspective to future healthcare professionals when addressing sexual diversity topics in order to reduce homophobia.

## Data availability statement

The raw data supporting the conclusions of this article will be made available by the authors, without undue reservation.

## Ethics statement

The studies involving human participants were reviewed and approved by Shanghai New York University Research Compliance Office. Written informed consent for participation was not required for this study in accordance with the national legislation and the institutional requirements.

## Author contributions

PS and QG contributed to the conception and design of the study. QG conducted data collection, organized the database, performed the statistical analysis, and wrote the first draft of the manuscript. All authors contributed to the manuscript revision and read and approved the submitted version.

## Conflict of interest

The authors declare that the research was conducted in the absence of any commercial or financial relationships that could be construed as a potential conflict of interest.

## Publisher’s note

All claims expressed in this article are solely those of the authors and do not necessarily represent those of their affiliated organizations, or those of the publisher, the editors and the reviewers. Any product that may be evaluated in this article, or claim that may be made by its manufacturer, is not guaranteed or endorsed by the publisher.

## Footnotes

^1^ www.credamo.com
